# Low LDL-Cholesterol and Hemorrhagic Risk: Mechanistic Insights and Clinical Perspectives

**DOI:** 10.3390/ijms26125612

**Published:** 2025-06-11

**Authors:** Carmine Siniscalchi, Manuela Basaglia, Tiziana Meschi, Egidio Imbalzano, Francesca Futura Bernardi, Alessandro Perrella, Ugo Trama, Angelica Passannanti, Pierpaolo Di Micco, Concetta Schiano

**Affiliations:** 1 Internal Medicine Department, Parma University Hospital, 43120 Parma, Italy; csiniscalchi84@gmail.com (C.S.); mbasaglia80@gmail.com (M.B.); tiziana.meschi@unipr.it (T.M.); 2 Department of Clinical and Experimental Medicine, University of Messina, 98122 Messina, Italy; egidio.imbalzano@unime.it; 3 Coordination of the Regional Health System, General Directorate for Health Protection, Department of Experimental Medicine, University of Campania Luigi Vanvitelli, 80131 Naples, Italy; francescafutura.bernardi@regione.campania.it; 4 UOC Emerging Infectious Disease and High Contagiousness, AORN Ospedale dei Colli, PO D. Cotugno, 80131 Naples, Italy; alessandro.perrella@ospedaledeicolli.it; 5 Directorate-General for Health Protection, Campania Region, Coordination of the Regional Health System, General Directorate for Health Protection, 80131 Naples, Italy; ugo.trama@regione.campania.it; 6 UOC Division of Clinical Immunology, Immunohematology, Department of Internal Medicine and Specialistics, University of Campania Luigi Vanvitelli, 80138 Naples, Italy; angelica.passannanti@policlinico-napoli.it; 7 AFO Medicina, PO Santa Maria delle Grazie, Pozzuoli Naples Hospital 2 Nord, 80078 Naples, Italy; 8 Department of Advanced Medical and Surgical Sciences (DAMSS), University of Campania Luigi Vanvitelli, 80138 Naples, Italy; concetta.schiano@unicampania.it

**Keywords:** atherosclerosis, cholesterol, hemostasis, personalized medicine, vascular integrity

## Abstract

Low-density lipoprotein cholesterol (LDL-C) plays a central role in lipid metabolism and is a well-established therapeutic target for the prevention of atherosclerotic cardiovascular diseases (CVDs). In recent years, increasingly aggressive lipid-lowering strategies have been adopted to achieve ultra-low LDL-C concentrations (<55 mg/dL or even <30 mg/dL) in high-risk patients. While the benefits of LDL-C reduction in lowering the incidence of myocardial infarction and ischemic stroke are well documented, emerging clinical evidence has raised concerns about a potential association between very low LDL-C levels and an increased risk of bleeding, particularly hemorrhagic stroke and gastrointestinal hemorrhage. This review critically examines the molecular mechanisms by which reduced LDL-C levels may influence the hemostatic system and vascular integrity. It explores the complex interplay between cholesterol availability and platelet function, endothelial barrier stability, and coagulation pathways. In addition, we assess experimental and clinical studies supporting this association and discuss how these findings may inform risk stratification and personalized lipid-lowering strategies. A deeper understanding of the biological basis of this paradoxical risk is essential for achieving a safe, balanced, and effective approach to cardiovascular prevention.

## 1. Introduction

Over the past two decades, lowering low-density lipoprotein cholesterol (LDL-C) has become a cornerstone in the prevention and management of cardiovascular diseases (CVDs). Landmark trials such as SPARCL, JUPITER, FOURIER, and ODYSSEY OUTCOMES have shown that aggressive LDL-C reduction decreases the risk of myocardial infarction and ischemic stroke. However, emerging clinical evidence from these and other studies has highlighted an association between very low LDL-C levels—typically below 50 mg/dL—and an increased incidence of bleeding complications, particularly hemorrhagic stroke and gastrointestinal bleeding. These clinical signals raise important questions regarding the safety thresholds of lipid-lowering strategies.

Low-density lipoprotein cholesterol (LDL-C) plays a central role in lipid metabolism and is a well-established therapeutic target in the prevention of atherosclerotic cardiovascular diseases (CVDs). In recent years, increasingly aggressive lipid-lowering strategies have been implemented to achieve ultra-low LDL-C concentrations (<55 mg/dL or even <30 mg/dL) in high-risk patients. While the benefits of LDL-C reduction in decreasing the incidence of myocardial infarction and ischemic stroke are well documented, emerging clinical evidence has raised concerns about a possible association between very low LDL-C levels and an elevated risk of bleeding [1].

To achieve these ambitious targets, clinicians now routinely combine statins, with adjunctive therapies such as ezetimibe, proprotein convertase subtilisin/kexin type 9 (PCSK9) inhibitors (e.g., evolocumab, alirocumab), and small interfering RNA (siRNA)-based agents like inclisiran. These pharmacologic advances have made previously unattainable LDL-C concentrations a reality in clinical practice. However, the push toward ever-lower thresholds has raised important safety questions: Is there a physiological “floor” for LDL-C, below which unintended adverse effects emerge? If so, what are the underlying biological physiological mechanisms?

In addition to bleeding concerns, LDL-C lowering therapies are associated with other adverse drug reactions (ADRs), particularly idiosyncratic drug-induced liver injury (DILI). Statins, although generally safe, have been implicated in rare but serious hepatic events. PCSK9 inhibitors and inclisiran, despite having more favorable hepatic safety profiles, have also been associated with isolated cases of hepatotoxicity. Several case reports and pharmacovigilance studies have used the Roussel Uclaf Causality Assessment Method (RUCAM) to confirm the causality of DILI in patients on statin therapy, sometimes leading to treatment discontinuation or modification. Clinicians should be aware of these rare ADRs, especially in older adults, patients with polypharmacy, or those with pre-existing liver disease [2,3].

One of the most notable concerns is the observed association between low LDL-C levels and an elevated risk of bleeding. This relationship has been most consistently documented in the context of hemorrhagic stroke, with additional signals emerging for gastrointestinal and mucosal bleeding. Although initially dismissed by some as statistical noise or confounding by indication, a growing body of mechanistic and translational research suggests that this association may be biologically plausible. Cholesterol is not merely a lipid transporter, it plays a fundamental role in the structural and functional integrity of cell membranes, particularly in platelets and vascular endothelial cells. Cholesterol-rich lipid rafts are essential for membrane signaling, protein localization, and barrier function. In the vascular system, LDL-derived cholesterol contributes to the balance of pro-thrombotic and anti-thrombotic pathways. Its pharmacologic or genetic depletion can impair platelet function compromise endothelial resilience, and potentially disrupt the delicate equilibrium of the coagulation cascade.

This review aims to summarize both the clinical evidence and the mechanistic rationale linking extremely low LDL-C to increased bleeding risk, focusing on endothelial integrity, platelet function, and coagulation balance. We examine cholesterol’s role in platelet aggregation to endothelial permeability and coagulation factor activity. Additionally, we assess clinical and translational studies that support this association and explore how these insights may inform personalized risk–benefit assessments in lipid-lowering therapy. A nuanced understanding of this emerging paradox is essential for refining therapeutic targets and ensuring safe, individualized cardiovascular prevention strategies.

## 2. Cholesterol and Platelet Biology: From Lipid Rafts to Aggregation

Platelets are anucleate cytoplasmic fragments derived from megakaryocytes, primarily tasked with preserving hemostasis through a coordinated sequence of adhesion, activation, aggregation, and thrombus formation in response to vascular injury. While platelet function has traditionally been studied through the lens of glycoprotein receptors, intracellular signaling cascades, and granule release, growing attention has been directed towards the critical role of lipid composition—particularly cholesterol—in modulating platelet physiology. Cholesterol constitutes approximately 30–40% of the lipid content of the platelet plasma membrane and is highly concentrated within specialized membrane microdomains known as lipid rafts. These cholesterol-enriched regions serve as organizing platforms for clustering key receptors, G-proteins, and signaling intermediates that orchestrate platelet activation. Notably, lipid rafts facilitate the spatial assembly of integrins (e.g., GPIIb/IIIa), purinergic receptors (e.g., P2Y12), and Fc receptors, all of which are indispensable for effective thrombus formation [4,5,6].

Experimental depletion of membrane cholesterol using agents such as methyl-β-cyclodextrin has been shown to disrupt lipid raft architecture, impair calcium mobilization, and blunt responses to classical agonists including ADP, thrombin, and collagen [7]. Similar impairments have been observed in animal models of hypocholesterolemia and in vitro systems exposed to LDL-depleted serum, further supporting the role of LDL-derived cholesterol as a functional modulator of platelet responsiveness [8,9,10].

Although initially dismissed by some as statistical noise or confounding by indication, a growing body of mechanistic and translational research suggests that this association may be biologically plausible. Cholesterol is not merely a lipid transporter, it plays a fundamental role in the structural and functional integrity of cell membranes, particularly in platelets and vascular endothelial cells [4,5,6]. Cholesterol-rich lipid rafts are essential for membrane signaling, protein localization, and barrier function. In the vascular system, LDL-derived cholesterol contributes to the balance of pro-thrombotic and anti-thrombotic pathways. Its pharmacologic or genetic depletion can impair platelet function [7,8,9,10], compromise endothelial resilience, and potentially disrupt the delicate equilibrium of the coagulation cascade [11,12,13].

Statins, through inhibition of HMG-CoA reductase, reduce intracellular cholesterol synthesis and thereby alter the lipid composition of the platelet membrane. Clinical studies have documented decreased platelet aggregation and lower thromboxane A2 production in statin-treated patients—findings that contribute to the drugs’ atheroprotective properties but also raise concerns about the potential for over-suppression of hemostatic capacity [11,12,13].

PCSK9 inhibitors and inclisiran, although mechanistically distinct from statins, also lead to marked reductions in circulating LDL-C. While they do not directly inhibit cholesterol biosynthesis, the resulting depletion of plasma LDL may limit the cholesterol reservoir available for membrane maintenance, particularly in cells with limited capacity for de novo synthesis [14,15,16].

Emerging data from lipidomics and membrane biophysics studies suggest that profound LDL-C lowering can alter the biophysical properties of the platelet membrane, including stiffness, surface charge, and lipid raft dynamics. These modifications may impair platelet adhesion under shear stress and reduce granule secretion in response to stimulation, with potential clinical implications [17,18,19].

In summary, cholesterol is far more than a passive structural element in platelet biology; it is a dynamic regulator of membrane organization and signal transduction. Profound reductions in LDL-C, while beneficial in mitigating atherothrombotic risk, may inadvertently impair platelet reactivity, thereby increasing bleeding susceptibility—particularly in patients receiving concurrent antiplatelet or anticoagulant therapies.

## 3. Endothelial Integrity and LDL-C: Mechanisms of Microvascular Fragility

The endothelium is a dynamic and multifunctional interface between the bloodstream and vascular tissues, functioning both as a physical barrier and as a signaling hub that regulates vascular tone, permeability, and immune surveillance. Its structural integrity is essential for preventing spontaneous bleeding and for maintaining selective permeability to solutes and cells.

Cholesterol plays a pivotal role in determining endothelial cell membrane composition and function. It contributes to the organization of tight junction proteins, supports the formation of caveolae—cholesterol- and caveolin-rich lipid microdomains—and modulates both transcellular and paracellular transport pathways [20,21,22]. Disruption of cholesterol homeostasis within endothelial cells has been associated with increased vascular permeability and heightened susceptibility to mechanical stress.

Animal studies have consistently shown that cholesterol depletion compromises endothelial tight junctions, leading to increased leakage of plasma proteins and erythrocytes into the interstitium [23]. In models of genetic hypocholesterolemia or dietary cholesterol restriction, endothelial barrier dysfunction is further associated with upregulation of pro-inflammatory cytokines and matrix metalloproteinases (MMPs), which promote degradation of extracellular matrix components and basement membranes [24,25].

At the molecular level, cholesterol deficiency reduces membrane fluidity and disrupts the proper localization of critical tight junction proteins such as occludin, claudins, and VE-cadherin. These proteins are essential for the formation of the zonula occludens, which ensures endothelial cohesion and restricts paracellular transport [26]. In conditions of very low LDL-C, reduced cholesterol incorporation into endothelial membranes may impair junctional assembly and maintenance, predisposing to microvascular fragility.

Additionally, endothelial nitric oxide synthase (eNOS), a key enzyme responsible for vasodilation and anti-thrombotic signaling, is localized within caveolae. Cholesterol depletion disrupts caveolar architecture, diminishing eNOS activity and reducing nitric oxide (NO) bioavailability, thereby contributing to endothelial dysfunction [27,28]. Given that NO inhibits both platelet adhesion and leukocyte recruitment, its reduction not only compromises vascular tone but also increases the risk of thrombosis or, paradoxically, uncontrolled hemorrhage due to dysregulated vascular reactivity.

Clinical imaging studies corroborate these biological findings. Cerebral microbleeds, detected by susceptibility-weighted MRI sequences, are more prevalent in individuals with low LDL-C levels, particularly among older adults and those on long-term statin therapy [29,30]. These microbleeds typically occur in deep or lobar brain regions and are regarded as markers of cerebral small vessel disease, a well-established risk factor for intracerebral hemorrhage (ICH).

Moreover, histopathological analyses of cerebral and gastrointestinal vasculature in patients with hemorrhagic complications have revealed characteristic thinning of vessel walls, discontinuity of the endothelium, and degradation of the subendothelial matrix—features that may be exacerbated under conditions of sustained low-cholesterol availability [31,32,33].

In conclusion, while LDL-C is a well-known contributor to atherogenesis, it also plays a crucial role in preserving vascular structural integrity. Excessive lowering of cholesterol levels can impair endothelial function, compromise tight junctions, reduce NO signaling, and increase vulnerability to mechanical and inflammatory stressors. These alterations may help explain the increased bleeding risk observed in patients with very low LDL-C levels [34], particularly within high-shear, small-caliber vessels such as those in the brain and gastrointestinal tract. Clinical implications and translational perspectives are summarized in Table 1.

The coagulation system functions as a finely regulated cascade that responds to vascular injury by promoting thrombin generation, fibrin formation, and clot stabilization. Traditionally considered separate from lipid metabolism, emerging evidence suggests that cholesterol levels—particularly LDL-C—can significantly influence the balance between pro-thrombotic and anti-thrombotic pathways.

LDL-C engages in several indirect yet biologically relevant interactions with components of the coagulation cascade. For example, low LDL-C levels have been associated with reduced concentrations of key procoagulant factors, such as factor VII and fibrinogen, alongside increased activity of natural anticoagulants, including tissue factor pathway inhibitor (TFPI) and antithrombin III [35,36]. Collectively, these alterations may shift the hemostatic balance towards a hypocoagulable state, particularly when combined with antithrombotic therapies or pre-existing bleeding tendencies.

At the hepatic level, cholesterol-rich membranes play a central role in the biosynthesis and secretion of multiple coagulation proteins. LDL-C may also regulate hepatic expression of coagulation factors through sterol response element-binding proteins (SREBPs) and nuclear receptors such as liver X receptor (LXR) and farnesoid X receptor (FXR), which function as metabolic sensors tightly linked to hepatic lipid homeostasis [37,38]. Consequently, when LDL-C levels fall below physiological thresholds, synthesis of essential coagulation factors may be impaired, reducing thrombin generation potential.

In addition to hepatic effects, LDL-C modulates the expression and activity of tissue factor (TF), a key initiator of the extrinsic coagulation pathway. In vitro studies have shown that cholesterol depletion in monocytes and endothelial cells diminishes TF expression in response to inflammatory stimuli such as interleukin-1β or lipopolysaccharide (LPS) [39]. Moreover, lipid rafts in monocytes facilitate TF clustering and its interaction with factor VIIa, enhancing its procoagulant function. Thus, cholesterol depletion may impair TF-mediated coagulation under both physiological and pathological conditions [40].

Furthermore, lipoproteins—including LDL—can bind and neutralize LPS and other pro-inflammatory mediators, thereby dampening innate immune activation of the coagulation cascade. Low LDL-C levels may compromise this buffering capacity, enabling unchecked LPS–TLR4 signaling that contributes to endothelial injury and consumptive coagulopathy, potentially underlying bleeding in septic or inflammatory states [41,42].

Clinical evidence supports these mechanistic insights. Hypocholesterolemia has been associated with prolonged prothrombin time (PT) and reduced thrombin generation in both healthy individuals and patients with liver disease, malignancy, or critical illness [43,44]. These findings suggest that LDL-C is not merely a marker of nutritional or inflammatory status, but also an active modulator of hemostatic function.

Additionally, some lipid-lowering therapies may influence coagulation independently of their effects on LDL-C. For example, statins exert pleiotropic effects including attenuation of platelet activity, downregulation of TF expression, and modulation of fibrinolysis. While generally beneficial in atherosclerotic contexts, these effects may synergistically increase bleeding risk in predisposed individuals—particularly when LDL-C falls into the extreme hypocholesterolemic range [45,46].

In summary, the coagulation cascade does not operate in isolation from lipid metabolism. Profound reductions in LDL-C may impair hepatic production of coagulation factors, disrupt TF signaling, and interfere with immune–coagulation cross-talk. While often clinically silent, these alterations may become significant in the setting of anticoagulation, surgery, trauma, or spontaneous microvascular injury, predisposing to bleeding complications.

## 4. Clinical Evidence Linking Low LDL-C to Hemorrhagic Outcomes

While mechanistic and preclinical studies suggest that very low LDL-C levels may impair platelet aggregation, compromise endothelial integrity, and disrupt coagulation balance, these findings are increasingly supported by real-world clinical outcomes. Over the past 15 years, several randomized controlled trials and large-scale observational studies have consistently reported an association between profoundly reduced LDL-C levels and an elevated risk of bleeding events, particularly intracerebral and gastrointestinal hemorrhages. Although most cardiovascular outcome trials were not originally designed to evaluate bleeding risk as a primary endpoint, notable safety signals have nonetheless emerged from subgroup analyses and post hoc evaluations, underscoring the need for careful consideration of bleeding risk in patients achieving very low LDL-C levels.

### 4.1. Hemorrhagic Stroke

The most robust clinical evidence linking low LDL-C to increased bleeding risk comes from the SPARCL (Stroke Prevention by Aggressive Reduction in Cholesterol Levels) trial, which enrolled 4731 patients with a recent ischemic stroke or transient ischemic attack and no known coronary heart disease. Participants were randomized to receive high-dose atorvastatin (80 mg daily) or placebo. While atorvastatin significantly reduced the incidence of recurrent ischemic stroke, it was associated with a 66% increased risk of hemorrhagic stroke (hazard ratio [HR]: 1.66; 95% CI: 1.08–2.55) [47].

Subsequent meta-analyses have reinforced these concerns. A pooled analysis of randomized 26 statin trials involving over 100,000 participants identified a U-shaped relationship between LDL-C and hemorrhagic stroke risk, with the lowest risk observed at LDL-C concentrations of 90–110 mg/dL and a significantly increased risk when levels fell below 50 mg/dL [48,49].

Similarly, in the J-STARS trial, conducted in a lower-risk Japanese population, it was found that statin therapy did not reduce overall stroke incidence, but was associated with a higher rate of hemorrhagic events, particularly among participants with low baseline LDL-C [50].

These data findings have fueled ongoing debate about the safety of aggressive LDL-C lowering, particularly in individuals with cerebral small vessel disease or pre-existing cerebral microbleeds. Supporting this concern, several MRI-based observational studies have demonstrated that low LDL-C levels correlate with an increased burden of cerebral microbleeds, which are recognized as potent predictors of future intracerebral hemorrhage [51,52,53].

### 4.2. Gastrointestinal and Mucosal Bleeding

Although less striking than in the association with hemorrhagic stroke, an increased risk of gastrointestinal bleeding (GIB) has also been observed and reported in individuals with low LDL-C levels. In the JUPITER trial, which assessed the effects of rosuvastatin in healthy individuals with elevated high-sensitivity C-reactive protein (hsCRP), a modest but statistically significant increase rise in GIB incidence was observed in the rosuvastatin group compared to placebo (1.2% vs. 0.9%, *p* = 0.03), despite the study population having a low baseline risk of bleeding [54].

These findings have been corroborated by real-world evidence. A large retrospective cohort study using Taiwan’s National Health Insurance Research Database (NHIRD) analyzed data from over 30,000 statin users and found that LDL-C levels below 70 mg/dL were associated with a 25% higher risk of GIB, particularly in patients concurrently receiving antiplatelet therapy [55].

Furthermore, case reports and small-scale studies have documented increased mucosal bleeding, such as epistaxis, gingival bleeding, and hematuria, in patients with profoundly low LDL-C (<30 mg/dL). However, these bleeding manifestations are frequently underrecognized or inconsistently reported in larger registries and trials [56,57].

### 4.3. Meta-Analyses and Pooled Data

A 2021 meta-analysis encompassing over 300,000 patients from 16 randomized clinical trials and 14 observational studies reported that LDL-C levels below 50 mg/dL were associated with a 1.4- to 1.6-fold increased risk of major bleeding events. This association was particularly evident in individuals receiving concomitant antiplatelet or anticoagulant therapy [58]. Importantly, while the overall bleeding risk did not negate the cardiovascular benefits of aggressive LDL-C lowering in the general population, the risk became clinically significant in specific subgroups, namely older adults and those with a history of hemorrhagic events.

Similarly, pooled analysis from the FOURIER and ODYSSEY OUTCOMES trials, both large-scale studies of PCSK9 inhibitors, revealed that although the absolute incidence of bleeding events was low, there was a numerical increase in episodes of epistaxis, gastrointestinal, and retinal bleeding among patients achieving LDL-C levels below 30 mg/dL [15,16].

It is crucial to note that none of these LDL-C lowering trials were originally designed or powered to assess bleeding risk as a primary endpoint. This methodological limitation underscores the need for future prospective studies incorporating bleeding endpoints into the safety–efficacy assessment of intensive lipid-lowering strategies.

## 5. Implications for Clinical Practice and Future Research

### 5.1. Personalizing LDL-C Targets Based on Bleeding Risk

While intensive LDL-C lowering has consistently demonstrated cardiovascular benefits in both primary and secondary prevention, the potential for increased bleeding risk at extremely low LDL-C levels highlights the importance of personalized therapy. Clinical guidelines, such as those from the European Society of Cardiology (ESC) and American College of Cardiology/American Heart Association (ACC/AHA), currently recommend LDL-C targets <55 mg/dL for very high-risk patients, and even <40 mg/dL for those with recurrent events [1,2,3]. However, these guidelines do not yet incorporate bleeding risk as a modifying factor in target selection.

In clinical practice, healthcare providers must balance the ischemic benefits of aggressive LDL-C reduction with the potential for increased bleeding risk, especially in patients with risk-enhancing features such as the following:

- A history of intracerebral hemorrhage or cerebral microbleeds on MRI;
- Advanced age (>75 years);
- Concurrent use of anticoagulants or dual antiplatelet therapy;
- A low body mass index (BMI < 20 kg/m^2^);
- A history of gastrointestinal bleeding or peptic ulcer disease;
- Asian ethnicity, which may confer heightened bleeding sensitivity at low LDL-C levels [50,51,52,53,55,58].

For these patients, a more moderate LDL-C target, (e.g., 70–100 mg/dL) may present a safer compromised approach, particularly in cases complicated by polypharmacy or frailty. This personalized strategy allows for optimizing cardiovascular protection while minimizing bleeding risks.

### 5.2. Tailoring Lipid-Lowering Regimens and Co-Medications

In patients at risk of bleeding, selecting lipid-lowering agents with a favorable safety profile is crucial. For instance, combining a moderate-intensity statin with ezetimibe can effectively lower LDL-C levels without reaching excessively low thresholds. Alternatively, although PCSK9 inhibitors are highly effective, they may be introduced with reduced frequency or duration if bleeding risks arise during follow-up. Co-management with other specialists is often necessary, particularly for patients on anticoagulants or those with neurovascular comorbidities. Key strategies may include the following:

- Discontinuing dual-antiplatelet therapy after 12 months, unless indicated as absolutely necessary;
- Co-prescribing proton pump inhibitors for gastroprotection;
- Using bleeding risk calculators (e.g., HAS-BLED, PRECISE-DAPT) to guide the intensity and duration of therapy [59].

Routine laboratory monitoring, including platelet function assays and occult blood testing, can also aid in detecting subclinical bleeding before it becomes clinically apparent.

### 5.3. Knowledge Gaps and Research Priorities

Despite a growing body of evidence supporting the biological plausibility and clinical relevance of bleeding associated with low LDL-C, several key questions remain unanswered:

- What is the “threshold” LDL-C level at which bleeding risk significantly increases? While <50 mg/dL is commonly recognized as a general marker, individual susceptibility varies.
- How does the duration of exposure to very low LDL-C influence bleeding risk? Longitudinal cohort studies are needed to assess time-dependent effects.
- Are certain genetic variants associated with an increased risk of bleeding when LDL-C is low? Mendelian randomization and genome-wide association studies may offer valuable insights.
- Can we develop predictive models that integrate both ischemic and hemorrhagic risks for managing LDL-C? Such models could help personalize therapy beyond the current binary targets.

Future clinical trials focusing on LDL-C lowering, particularly those involving novel therapies like siRNA and gene editing, should incorporate bleeding as a pre-specified safety endpoint, stratified by baseline risk. Additionally, translational studies using molecular imaging, endothelial functional assays, and lipidomic profiling may help clarify the mechanistic pathways linking hypocholesterolemia to bleeding in various vascular territories.

## 6. Conclusions

Low-density lipoprotein cholesterol (LDL-C) has traditionally been considered a key modifiable risk factor for atherosclerotic cardiovascular disease (ASCVD), with intensive LDL-C lowering forming the cornerstone of prevention strategies. However, as lipid-lowering therapies become more potent and LDL-C targets are pushed to increasingly lower thresholds, accumulating evidence suggests that achieving extremely low LDL-C levels—particularly below 50 mg/dL—may entail unintended consequences, including an elevated risk of bleeding events. This risk appears most evident in the form of intracerebral and gastrointestinal hemorrhages and is likely mediated by a combination of interconnected molecular mechanisms.

At the cellular and molecular levels, cholesterol is integral to the structural and functional integrity of platelet membranes, endothelial barriers, and coagulation pathways. Profound LDL-C reduction can disrupt these processes by impairing platelet aggregation, destabilizing endothelial tight junctions, suppressing nitric oxide (NO) bioavailability, and attenuating thrombin generation capacity. Collectively, these alterations may shift the delicate hemostatic balance towards a bleeding-prone phenotype, particularly in susceptible populations such as elderly patients, individuals with a history of bleeding, or those receiving concomitant antithrombotic therapy.

Clinical data from randomized controlled trials, meta-analyses, and observational studies support a more nuanced approach when aiming for ultra-low LDL-C levels. While the atheroprotective benefits of aggressive LDL-C lowering remain robust, these must be carefully weighed against the emerging hemorrhagic risk. This evolving evidence base underscores the need for individualized risk stratification strategies, especially in populations with competing bleeding risks.

From a translational perspective, these insights open new avenues for precision lipidology. Future research should prioritize the development of integrative algorithms that combine traditional ischemic risk assessment with bleeding risk prediction, enabling clinicians to tailor LDL-C targets according to the overall vascular vulnerability of each patient. Moreover, the exploration of biomarkers reflecting endothelial integrity, platelet function, or coagulation competence in the context of lipid-lowering therapies may further refine clinical decision making.

In conclusion, the challenge in modern cardiovascular prevention is not to abandon ambitious LDL-C goals, but to implement them judiciously, ensuring that lipid management strategies optimize both efficacy and safety. This approach will require a paradigm shift towards precision medicine, where LDL-C reduction is embedded within a broader framework of vascular health maintenance, balancing ischemic and hemorrhagic outcomes. Table 2 summarizes the clinical evidence linking very low LDL-C levels to increased bleeding risk across different studies and populations.

**Figure 1:**
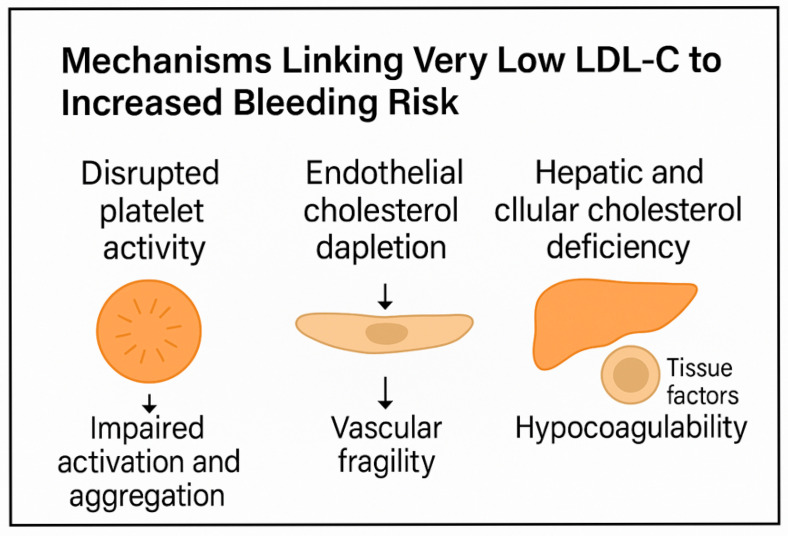
Schematic representation of the proposed mechanisms linking very low LDL-C levels to increased bleeding risk. Disruption of platelet lipid rafts impairs activation and aggregation. Endothelial cholesterol depletion weakens tight junctions and reduces NO production, promoting vascular fragility. Hepatic and cellular cholesterol deficiency alters coagulation factor synthesis and tissue factor expression, shifting the balance toward hypocoagulability.

These findings are summarized in Figure 1, which illustrates the multifaceted impact of very low LDL-C on platelet function, endothelial integrity, and coagulation balance. A better understanding of these mechanisms may help to personalize lipid-lowering therapy and avoid bleeding complications in at-risk populations.

## Figures and Tables

**Table 1:** Coagulation pathways and the impact of LDL-C on hemostatic balance.

Clinical Implications
○ While intensive LDL-C lowering remains a cornerstone for ASCVD prevention, emerging data suggest an association between extremely low LDL-C (<50 mg/dL) and increased bleeding risk, particularly intracerebral and gastrointestinal hemorrhage. ○ Special caution is warranted in high-risk groups, including older adults, patients with prior bleeding history, those on anticoagulant or antiplatelet therapy, and individuals with frailty or small vessel disease. ○ Current guidelines may benefit from incorporating bleeding risk stratification when considering aggressive LDL-C targets in selected patients.
**Translational perspectives**
○ Future lipid management strategies should move toward precision medicine, integrating both ischemic and hemorrhagic risk factors into personalized LDL-C goal setting. ○ Development of biomarkers reflecting endothelial integrity, platelet function, and coagulation capacity could aid in identifying patients at higher risk of bleeding during intensive lipid-lowering therapy. ○ Clinical trials and observational studies should systematically report bleeding outcomes alongside ischemic events to provide a more balanced evaluation of net clinical benefit in the era of very low LDL-C.

**Table 2:** Summary of key randomized clinical trials, observational studies, and meta-analyses evaluating the association between very low LDL-C levels and bleeding risk. The table highlights the type of study, population characteristics, bleeding outcomes reported, and relevant notes regarding concomitant therapies or specific subgroups at higher risk.

Study/Analysis	Population	LDL-C Target/Achieved	Type of Bleeding Observed	Key Findings	Reference
**SPARCL**	4731 patients with stroke or TIA	Median LDL-C: ~73 mg/dL with atorvastatin 80 mg	Intracerebral hemorrhage	66% increased risk of ICH (HR: 1.66; 95% CI: 1.08–2.55)	[47]
**Meta-analysis 2021**	>300,000 patients (16 RCTs + 14 observational studies)	LDL-C < 50 mg/dL	Major bleeding (various sites)	1.4–1.6-fold increased risk, especially with antithrombotic therapy	[58]
**FOURIER + ODYSSEY OUTCOMES (pooled)**	High CV risk patients treated with PCSK9 inhibitors	LDL-C < 30 mg/dL (achieved)	Epistaxis, GIB, retinal bleeding	Numerical increase in bleeding events, but low absolute incidence	[59]
**J-STARS (Japan)**	Patients with low baseline LDL-C at stroke risk	Already low baseline LDL-C	Intracranial bleeding	Increased hemorrhagic events, particularly in those with low initial LDL-C	[50]
**JUPITER**	17,802 healthy individuals with elevated hsCRP	Median LDL-C: ~55 mg/dL with rosuvastatin 20 mg	GIB	Increased GIB (1.2% vs. 0.9%, *p* = 0.03)	[54]
**NHIRD (Taiwan)**	>30,000 patients on statin therapy	LDL-C < 70 mg/dL	GIB	25% increased risk of GIB, particularly with concomitant antiplatelet agents	[55]

## Data Availability

Data supporting the reported results can be requested by email to the corresponding author.
